# The effect of bleaching applications on stained bulk-fill resin composites

**DOI:** 10.1186/s12903-022-02414-9

**Published:** 2022-09-10

**Authors:** Ayse Tugba Erturk-Avunduk, Esra Cengiz-Yanardag, Izgen Karakaya

**Affiliations:** 1 Department of Restorative Dentistry, Faculty of Dentistry, Mersin University, Çiftlikköy Campus, 33343 Yenişehir, Mersin, Türkiye; 2 Department of Restorative Dentistry, Faculty of Dentistry, European University of Lefke, TRNC, Mersin 10, Türkiye

**Keywords:** Bleaching, Bulk-fill, CIEDE2000, Color stability, Whiteness index

## Abstract

**Background:**

The structure of bulk-fill resin composites differs from that of their conventional counterparts, but how this difference affects the color stability of the former after staining and bleaching is unclear. Accordingly, this study was aimed at investigating color change in nine bulk-fill resin composites and one nanohybrid resin composite treated with hydrogen peroxide and carbamide peroxide after staining with tea, coffee, and red wine.

**Methods:**

Eighty specimens were prepared from each resins [Clearfil Majesty Posterior (CMP), SDR flow^+^ (SDR), Filtek^TM^Bulk-Fill Flowable Restorative (FBF), Reveal HD Bulk (RHD), Beautifil-Bulk Restorative (BBR), Tetric EvoCeram® Bulk Fill (TEC), SonicFill™2 (SF2), everX Posterior™ (eXP), X-tra base (XB), and Venus® Bulk Fill (VBF)]. Following baseline color measurements, the specimens were randomly divided into 4 groups according to immersion solutions and distilled water as the control. At the end of a 30-day test period, color measurements were repeated, and color change values (∆E_00_) were calculated. Each resin group was then divided into 2 subgroups (with 10 specimens per group) on the basis of bleaching agent (Opalescence Boost 40%, Opalescence PF 16%). Following bleaching application, ∆E_00_ and changes of whiteness (∆WI_D_1 = WI_D_bleaching-WI_D_baseline, ΔWI_D_2 = WI_D_bleaching-WI_D_staining) values were recorded. Two- and three-way analyses of variance and Tukey’s post hoc test were performed, with a P < 0.05 regarded as indicative of significance.

**Results:**

After immersion in distilled water, tea, and red wine, the highest ΔE_00_ values were observed in eXP (P < 0.05). Resin materials immersed in coffee and tea exhibited statistically higher ∆E_00_ values than those immersed in red wine except for eXP, TEC, and FBF (P < 0.05). For eXP, the highest ∆E_00_ values were recorded in distilled water. For TEC and FBF, there was no statistically significant difference among the immersion solutions and distilled water (P > 0.05). For all the resins and staining beverages, no statistically significant difference in ∆WI_D_1 and ∆WI_D_2 values were detected between bleaching agents (P > 0.05). All the ΔWI_D_1 values were above the whiteness perceptibility threshold.

**Conclusion:**

The bulk-fill materials were more resistant to discoloration and bleaching procedures than the conventional resin composites. Coffee and tea caused more staining than distilled water and red wine generally. The type of bleaching procedure had no effect on the whiteness of the tested materials.

## Background

The color harmony between resin composites and natural teeth has become a challenge for clinicians with the widespread use of esthetic restorative materials in recent years and the increasing interest of patients [1]. However, achieving this harmony may be difficult due to the shortcomings of the resin composite including porosities, inhomogeneity, lack of color stability, leakage and polymerization shrinkage which affect the esthetic features of these materials over time [2]. To overcome these disadvantages, the monomer chemistry and filler structure of resin composite have been consistently improved [3].

Incremental layering techniques have been suggested for the placement of conventional resin composites to increase the interaction between photo activators and curing light which reduces polymerization stress and provides a homogeneous degree of conversion throughout the material thickness [3, 4]. These techniques have some drawbacks such as being time consuming [3], having the risk of leading unbonded layers due to the air bubble entrapment and moisture contamination between increments [4], and void formation throughout the entire depth of large restorations [5]. To reduce the number of clinical steps and minimize the formation of internal and external marginal voids [6], bulk-fill resin composites which have been claimed to be placed as single-layer thicknesses of 4–6 mm in contrast to the commonly used conventional thickness of 2 mm were introduced [7, 8]. These newly developed materials have polymerization accelerators in their composition that decrease light curing time and allow increased depth of cure [7] which can mainly be associated to their higher translucency properties compared to conventional resins [8]. Although, the literature regarding the mechanical properties, depth of cure, marginal integrity and bond strength of bulk-fill resin composites are inconsistent, the vast majority of studies exhibited a relevant increase in depth of cure and decrease in polymerization shrinkage stress for bulk-fill resin composites when compared to conventional incrementally placed resin composites [7, 9].

Complex events occurring in the oral cavity throughout time may lead to changes in the color of resin composites within a certain period which affects the long-term success of the restorations [10]. The degree of discoloration which may be due to internal or external causes [11] is influenced by many factors such as water absorption, incomplete polymerization, oral hygiene, diet, the surface characteristics of the restoration and finishing and polishing procedures [12, 13]. The organic structure and filler particle properties of resin composites also have a direct effect on the surface roughness of the restoration and the tendency to external coloring [14]. Commonly consumed beverages, including tea, coffee, red wine, fruit juice, and cola have been reported to cause significant discoloration in resin composites [15].

As an effective and conservative approach, at-home and in-office bleaching techniques have been extensively used to remove the pigmentation and stains caused by intrinsic and extrinsic factors [16]. In both techniques, hydrogen peroxide (HP) and carbamide peroxide (CP) gels of varying concentrations have been applied [2]. Reactive oxygen species (ROS) released from the bleaching agents oxidize chromophores penetrated to the dental structure leading to the formation of smaller molecules, which reflects light more and so the teeth are perceived with lighter colors [16]. During bleaching procedures, ROS can also interact with the existing restorations, which explains why previous studies have intensively investigated the effects of bleaching agents on the physical, chemical, and optical properties of restorative materials [2, 12].

Several studies have analyzed the effects of HP and CP on resin composites [12, 17, 18] however, to the best of our knowledge, limited information has been derived as to the influence of in-office and at-home bleaching agents on the color stability of bulk-fill resin composites. Therefore, the aim of the present study was to analyze the color stability of 9 bulk-fill resin composites and a nanohybrid resin composite, staining effects of 3 beverages (tea, coffee, and red wine) and whiteness change of stained resin materials after at-home and in-office bleaching procedures. The null hypotheses of the present study were defined as: (1) There is no significant difference between the color stability of different bulk-fill resin composites and that of the nanohybrid resin composite. (2) No significant difference exists among the staining effectiveness of tea, coffee, and red wine. Finally, (3) No significant difference is found between the bleaching effects of at-home and in-office bleaching agents.

## Methods

### Materials

A nanohybrid [Clearfil Majesty Posterior (CMP)], and 9 bulk-fill resin composites [SDR flow ^+^ (SDR), Filtek™ One Bulk Fill Restorative (FBF), Reveal HD Bulk (RHD), Beautifil-Bulk Restorative (BBR), Tetric EvoCeram® Bulk Fill (TEC), SonicFill™ 2 (SF2), everX Posterior™ (eXP), X-tra base (XB), and Venus® Bulk Fill (VBF)] were analyzed in this study. The manufacturers, shades, and compositions of resin composites are presented in Table 1.

**Table 1:** Compositions of the composite resin materials

Product	Manufacturer	Shade	Classification	Composition		
				Monomer composition	Filler type	Filler amount (wt%/vol%)
Clearfil Majesty Posterior (CMP)	Kuraray Noritake Dental Inc.; Okayama, Japan	A2	Nanofilled posterior restorative	Bis-GMA, TEGDMA, and hydrophobic aromatic dimethacrylate	Glass ceramics, surface-treated alumina micro filler, silica	92/82
SDR™ Flow^+^ (SDR)	Dentsply, Konstanz, Germany	U	Bulk-Fill flowable composite	Modified UDMA, ethoxylated bisphenol-A-dimethacrylate (EBPADMA), TEGDMA, butylated hydroxytoluene (BHT), UV stabiliser, titanium dioxide and iron oxides, camphoroquinone	Ba-Al-F-B-Siglass and St-Al-F-Si-glass	70.5/47.4
Filtek^TM^Bulk-Fill Flowable Restorative (FBF)	3 M-Espe; St. Paul, MN, USA	A2	Bulk-Fill flowable composite	Bis-GMA, UDMA, Bis-EMA, Procrylat resins	Ytterbium trifluoride, Zirconia/silica	64.5/42.5
Reveal HD Bulk (RHD)	Bisco Inc.; Schaumburg, IL, USA	A2	Bulk-Fill Posterior Restorative	Urethane Dimethacrylate, BisGMA, 3-(Trimethoxysilyl) propyl-2-Methyl-2- Propenoic Acid, Tert-butyl Perbenzoate	Ytterbium Fluoride	Not obtained from company
Beautifil-Bulk Restorative (BBR)	Shofu Inc.; Kyoto, Japan	A	Bulk-Fill Posterior Restorative	Bis-GMA, UDMA, Bis-MPEPP, TEGDMA	S-PRG filler based on fluoroboroaluminosilicate glass, polymerization initiator	87/74.5
Tetric EvoCeram® Bulk Fill (TECBF)	Ivoclar Vivadent AG, Schaan, Liechtenstein	IV A	Bulk-Fill Posterior Restorative	Bis-GMA, UDMA	Ba-Al-Si glass, prepolymer filler (monomer, glass filler, and ytterbium fluoride), spherical mixed oxide	79–81/60–61
SonicFill 2 (SF 2)	Kerr Corp.; Orange, CA, USA	A2	Hybrid Bulk-Fill composite	Bis-GMA, TEGDMA, EBPDMA, Bis-EMA	Silicon dioxide, barium glass, Zirconium oxide	83.5/83
everX Posterior™ (eXP)	GC Corporation Tokyo, Japan	U	Short fiber Reinforced-Bulk-Fill Flowable Composite	Bis-GMA, TEGDMA, PMMA	Short E-glass fiber filler, barium glass, Hybrid filler fractions	74.2/53.6
X-tra base (XB)	Voco, Cuxhaven, Germany	U	Bulk-Fill Flowable Composite	MMA, Bis-EMA Aliphatic di-methacrylate (UDMA),	Barium glass ceramic, fumed silica	75/58
Venus® Bulk Fill (VBF)	Heraeus Kulzer GmbH, Hanau, Germany	U	Bulk-Fill Flowable Composite	UDMA, EBPDMA	Ba-Al-F-Si glass, YbF_3_ and SiO_2_	65/38

### Specimen preparation

**Fig. 1:**
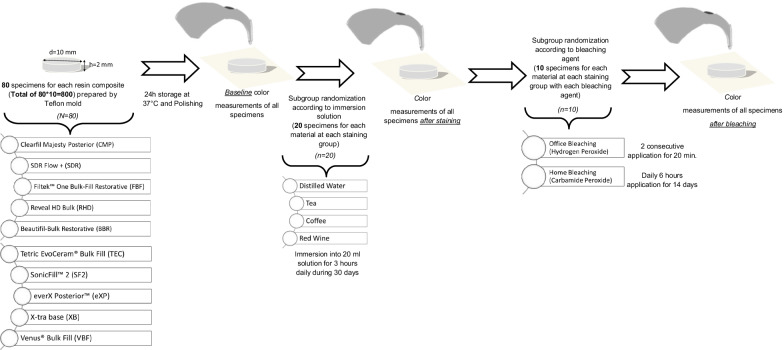
Schematic illustration of study design

A schematic illustration of specimen preparation and study design is presented in Fig. 1. The sample size number was calculated by using G*Power version 3.1.9.4 (Heinrich Heine, University of Düsseldorf, Düsseldorf, Germany) with a power of 90%. In the estimation, a supposed significance level of 0.05 and an effect size of 0.25 [19] were applied. Regarding the number of the materials (as 10), the number of the staining beverages (as 4) and the number of the bleaching agents (as 2), a total of 800 specimens were produced to obtain 10 specimens at the last subgroups of each material. Eighty specimens with 10 mm diameter and 2 mm thickness for each resin composite were fabricated by using teflon molds. To remove overlaid resin composite and achieve a smooth surface, a Mylar strip band (SS White Co.; Philadelphia, PA, USA) and a glass plate were lightly pressed onto the specimens. The polymerization of all the specimens were performed with a light-emitting diode (LED) curing light (Planmeca Lumion, Planmeca Oy, Helsinki, Finland) with an irradiance of 1070 mW/cm^2^ at a duration recommended by the manufacturer for each resin composite. A radiometer (Bluephase Meter II, Ivoclar Vivadent, Schaan, Liechtenstein) was used to verify the output intensity of the curing light before the polymerization of each group. 1-mm transparent polyester strip band was used to standardize the distance between the light unit and the sample. Metal rings were used to position the tip of the curing light unit placed on the resin composite before polymerization. All the specimens were polished with Super-Snap Rainbow Technique Kit (Shofu Inc., Kyoto, Japan) and One Gloss Polishing Kit (Shofu Inc., Kyoto, Japan). A digital caliper (N48AA, Maplin Electronics, UK) was used to control the final thickness of the specimens to 2 ± 0.1 mm. For post-polymerization, the specimens were immersed in distilled water at 37 °C for 24 h.

### Color measurement

The baseline (T_0_) and after staining (T_1_) color measurements of the specimens were performed by using a digital spectrophotometer (Vita Easyshade V, Vita Zahnfabrik, Bad Säckingen, Germany) operated in restoration measurement mode. The probe of the spectrophotometer device with a diameter of 5 mm was placed in the center of the specimens. After the device was calibrated in accordance with the manufacturer’s recommendations, the measurement of “L, C, and H” values were performed 3 times for each resin specimen on non-reflective white surface to eliminate interference form background light. These parameters were then converted into L*, a*, and b* values following the instructions on the website [20]. Afterwards, the average values of L*, a*, and b*were recorded. Following each 9 measurements, the spectrophotometer was re-calibrated based on the instructions of the manufacturer.

### Staining procedure

After the baseline color measurements were completed, the specimens were randomly divided into 4 groups (n = 20) according to the staining beverages and distilled water. The specimens were immersed in 20 ml of tea (Dogus Black Label Tea, Rize, Turkey) prepared as 2 g of tea in 300 ml of hot water, 20 ml of coffee (Nescafe Gold, Nestle, Istanbul, Turkey) prepared as 5 g of coffee in 300 ml of hot water, 20 ml of red wine (Yakut, Kavaklıdere, Ankara, Turkey) and 20 ml of distilled water for 3 h a day at room temperature over a 30-day staining period [21]. All the staining beverages were re-prepared daily. Distilled water was used as a control to evaluate intrinsic color changes within the restorative materials. After 3 h of immersion, the specimens were removed from the staining beverages, washed with distilled water, dried and all the resin specimens were kept in 20 ml distilled water for the rest of 24-h period. When 30 days of immersion were completed, the color measurements were repeated. To determine color differences after staining, ΔE_00_ (T_1-_ T_0_) values were calculated using the following equation [22]:

${\mathrm{\Delta E}_{\mathrm{00 }} = [(\frac{\Delta \mathrm{L}}{{\mathrm{k}}_{\mathrm{L }}{\mathrm{S}}_{\mathrm{L}}})}^{2 }+ {(\frac{\Delta \mathrm{C}}{{\mathrm{k}}_{\mathrm{C }}{\mathrm{S}}_{\mathrm{C}}})}^{2 }+{(\frac{\Delta \mathrm{H}}{{\mathrm{k}}_{\mathrm{H }}{\mathrm{S}}_{\mathrm{H}}})}^{2 }+{\mathrm{R}}_{\mathrm{T}}{(\frac{\Delta \mathrm{C}}{{\mathrm{k}}_{\mathrm{C }}{\mathrm{S}}_{\mathrm{C}}})(\frac{\Delta \mathrm{H}}{{\mathrm{k}}_{\mathrm{H }}{\mathrm{S}}_{\mathrm{H}}})}^{ }]^{1/2 }$where ΔL, ΔC, and ΔH denote lightness, chroma, and hue differences, respectively, which were calculated on the basis of the difference between the baseline and final color measurements. Weighting functions S_L_, S_C_, and S_H_ were incorporated into the formula to overcome the irregularities occurring in the CIE L*a*b system [22]. R_T_, which is the rotation function, fits chromatic differences in the blue region, thereby improving the performance of a color difference equation [23]. Parametric factors k_L_, k_C_, and k_H_ were adjusted according to different viewing parameters and experimental conditions. In this study, CIEDE2000 (1:1:1) was used, where k_L_ = k_C_ = k_H_ = 1 [17]. Finally, color changes were analyzed on the basis of an acceptability threshold of 50:50% (AT:ΔE_00_ = 1.8) and a perceptibility threshold of 50:50% (PT:ΔE_00_ = 0.8) for all the resin materials as recommended by the International Organization for Standardization [22, 24, 25].

### Bleaching procedure

After the second round of color measurements, the specimens of each staining group were separated as 2 subgroups (n = 10) depending on the bleaching agent applied. Two bleaching systems, including in-office (Opalescence Boost 40%) and at-home bleaching agent (Opalescence PF 16%) (Ultradent, South Jordan, Utah, USA) were evaluated in this study. For the in-office bleaching procedure, equal amounts of 40% HP bleaching gel were uniformly dispersed with an applicator (Ivoclar Vivadent, Schaan, Liechtenstein) onto the same surface areas of the specimens. HP was left to stand on the specimens for 20 min and activated by a micro-brush every 5 min, according to the instructions of the manufacturer. HP gel (40%) was re-applied to areas that had thinned or needed replenishing. As recommended by the manufacturer, this entire procedure was repeated one more for 20 min within the same session. For the at-home bleaching procedure, 16% CP gel was applied with a syringe (Beybi Plastik Fab. San. AS, Istanbul, Turkey) to standardize the amount of gel and uniformly dispersed with a cotton applicator on the same surface areas of the specimens. CP was left to stand on the surface for 6 h per day at room temperature for 14 days, according to the manufacturer’s recommendations. In both HP and CP applications, the specimens were washed under high pressure distilled water and dried using airflow and blotting paper after exposure to the bleaching agents. All the specimens were then immersed in 20 ml fresh distilled water in a period of 14 days when not exposed to the bleaching agent for the duration of the experiment [26]. After all the groups were bleached, the color values were re-measured as mentioned for the staining procedure. For the assessment of the whiteness change before and after bleaching application, a new CIELAB space-based Whiteness Index for Dentistry (WI_D_) was performed with following formula [27]:

$${\mathrm{WI}}_{D}=0.511{L}^{*}-2.324{a}^{*}-1.100{b}^{*}$$

Changes (ΔWI_D_) of tooth whiteness was calculated as follows:

$$\Delta {\text{WI}}_{{\text{D}}} {1} = {\text{WI}}_{{\text{D}}} \left( {{\text{bleaching}}} \right) - {\text{WI}}_{{\text{D}}} \left( {{\text{baseline}}} \right)$$

$$\Delta {\text{WI}}_{{\text{D}}} {2} =_{{}} {\text{WI}}_{{\text{D}}} \left( {{\text{bleaching}}} \right) - {\text{WI}}_{{\text{D}}} \left( {{\text{staining}}} \right)$$

For whiteness change analysis, the 50:50% whiteness perceptibility threshold of 0.72 units (WPT = 0.72) and the 50:50% whiteness acceptability threshold of 2.60 units (WAT = 2.60) were adopted [28].

### Statistical analyses

Statistical analyses were performed using the Statistical Package for the Social Sciences (SPSS Inc., Chicago, IL, USA). The Shapiro–Wilk test was conducted to analyze the normality of the ΔE_00_ and ΔWI_D_ data. Two-way analysis of variance (ANOVA) was used to detect the significance in terms of staining beverages. Three-way ANOVA was employed to indicate the influence of bleaching agents on color changes among the restorative materials in staining beverages and distilled water. For pairwise comparisons, the Tukey’s post-hoc test was performed with 95% confidence intervals to determine differences among the groups. P = 0.05 was considered as the level of statistical significance.

## Results

**Fig. 2:**
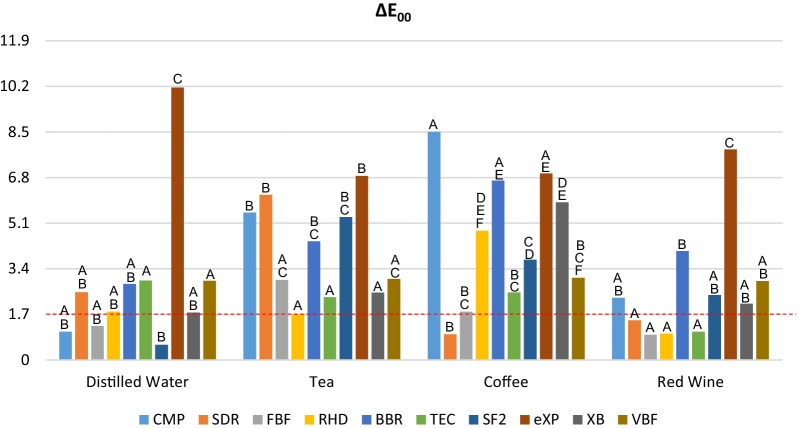
Avarage ΔE_00_ values of resin composite materials after staining procedure. The capital letters indicate the difference between the restorative materials. *CMP* Clearfil Majesty Posterior, *SDR* SDR™ Flow^**+**^, *FBF* Filtek™ Bulk-Fill Flowable Restorative, *RHD* Reveal HD Bulk, *BBR* Beautifil-Bulk Restorative, *TEC* Tetric EvoCeram® Bulk Fill, *SF2* SonicFill 2, *eXP* everX Posterior™, *XB* X-tra base, *VBF* Venus® Bulk Fill, *HP* hydrogen peroxide, *CP* carbamide peroxide

The mean ΔE_00_ values and standard deviations of the resin composites after immersion in the staining beverages are shown in Table 2. The intragroup comparison of the resin composite materials immersed in the staining solutions is schematized in Fig. 2. With regard to the color difference measurements, the highest ΔE_00_ values were detected in the eXP group, regardless of beverage type, with significant differences among the restorative materials (P < 0.05). Resin materials immersed in coffee and tea exhibited statistically higher ∆E_00_ values than those immersed in red wine except for eXP, TEC and FBF (P < 0.05). For TEC and FBF, there was no statistically significant difference among the staining solutions (P > 0.05). For eXP, distilled water showed statistically higher ΔE_00_ values than coffee and tea. Intragroup comparison of ΔE_00_ values of each material revealed that (Fig. 2), in distilled water and red wine, eXP showed the statistically highest ∆E_00_ values among the resin composites. Imperceptible ΔE_00_ values (PT < 0.8) were found only in SF2 distilled water group. For tea staining group, ΔE_00_ values of all resin composites are above the acceptability threshold. For coffee staining group, ∆E_00_ values of CMP, BBR and eXP are statistically higher than other resin composites (P > 0.05).

**Table 2:** The mean ΔE_00_ (T_1-_ T_0_) values ± standard deviations after staining procedure

ΔE_00_	Distilled water	Tea	Coffee	Red wine
CMP	1.06 ± 0.68^c^	*5.50 ± 3.71^b^	*8.50 ± 2.45^a^	*2.32 ± 0.89^c^
SDR	*2.53 ± 0.66^b^	*6.16 ± 3.60^a^	0.96 ± 0.58^b^	1.48 ± 1.29^b^
FBF	1.27 ± 0.44	*2.99 ± 2.85	*1.80 ± 1.85	0.94 ± 0.68
RHD	*1.80 ± 0.49^b^	1.71 ± 1.70^b^	*4.82 ± 2.73^a^	0.98 ± 0.42^b^
BBR	*2.83 ± 0.75^b^	*4.42 ± 2.99^a.b^	*6.69 ± 3.68^a^	*4.06 ± 1.89^b^
TEC	*2.96 ± 0.71	*2.34 ± 1.41	*2.52 ± 2.17	1.06 ± 0.47
SF2	0.57 ± 0.19^c^	*5.33 ± 2.24^a^	*3.74 ± 0.84^a.b^	*2.42 ± 1.37^b^
eXP	*10.16 ± 1.77^a,b^	*6.86 ± 1.82^c^	*6.95 ± 1.81^c^	*7.85 ± 1.37^b,c^
XB	1.77 ± 0.30^b^	*2.51 ± 1.69^b^	*5.88 ± 2.96^a^	*2.10 ± 1.26^b^
VBF	*2.95 ± 1.52	*3.02 ± 3.01	*3.06 ± 2.35	*2.94 ± 1.35

Lower letters indicate the difference throughout a row. (p < .05). *ΔE_00_ > AT (1.8)

*CMP* Clearfil Majesty Posterior, *SDR* SDR™ Flow^+^, *FBF* Filtek^TM^Bulk-Fill Flowable Restorative, *RHD* Reveal HD Bulk, *BBR* Beautifil-Bulk Restorative, *TEC* Tetric EvoCeram® Bulk Fill, *SF2* SonicFill 2, *eXP* everX Posterior™, *XB* X-tra base, *VBF* Venus® Bulk Fill

**Fig. 3:**
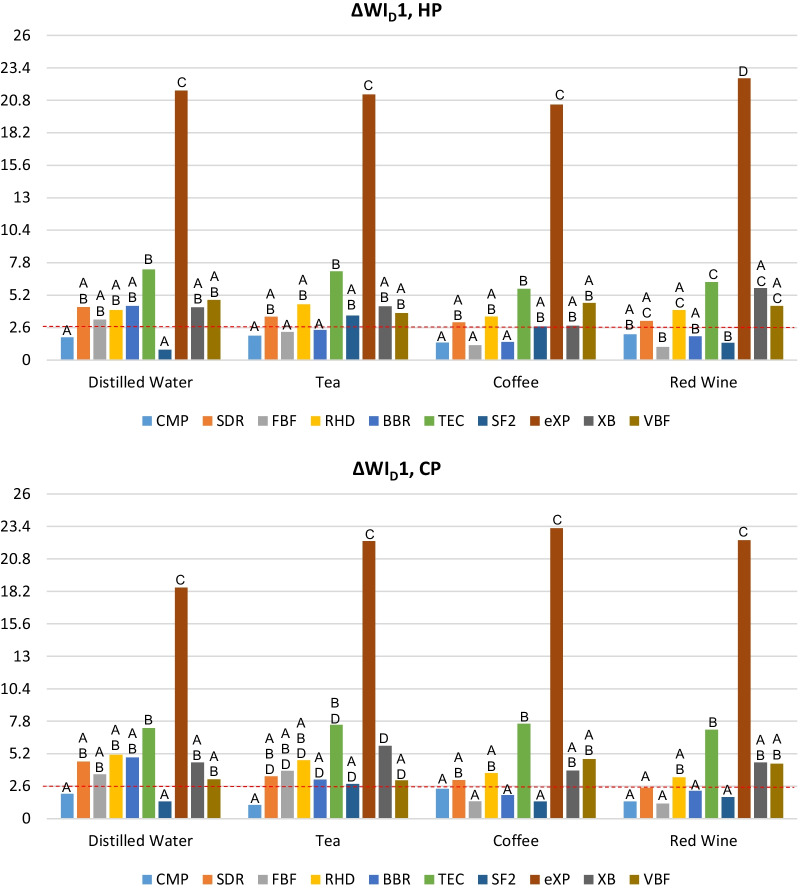
ΔWI_D_1 values of resin composite after two bleaching protocols. The capital letters indicate the difference between the restorative materials. ΔWI_D_1 = WI_D_ (bleaching) − WI_D_ (baseline), WPT = 0.72, WAT = 2.60, *CMP* Clearfil Majesty Posterior, *SDR* SDR™ Flow^+^, *FBF* Filtek™ Bulk-Fill Flowable Restorative, *RHD* Reveal HD Bulk, *BBR* Beautifil-Bulk Restorative, *TEC* Tetric EvoCeram® Bulk Fill, *SF2* SonicFill 2, *eXP* everX Posterior™, *XB* X-tra base, *VBF* Venus® Bulk Fill, *HP* hydrogen peroxide, *CP* carbamide peroxide

**Fig. 4:**
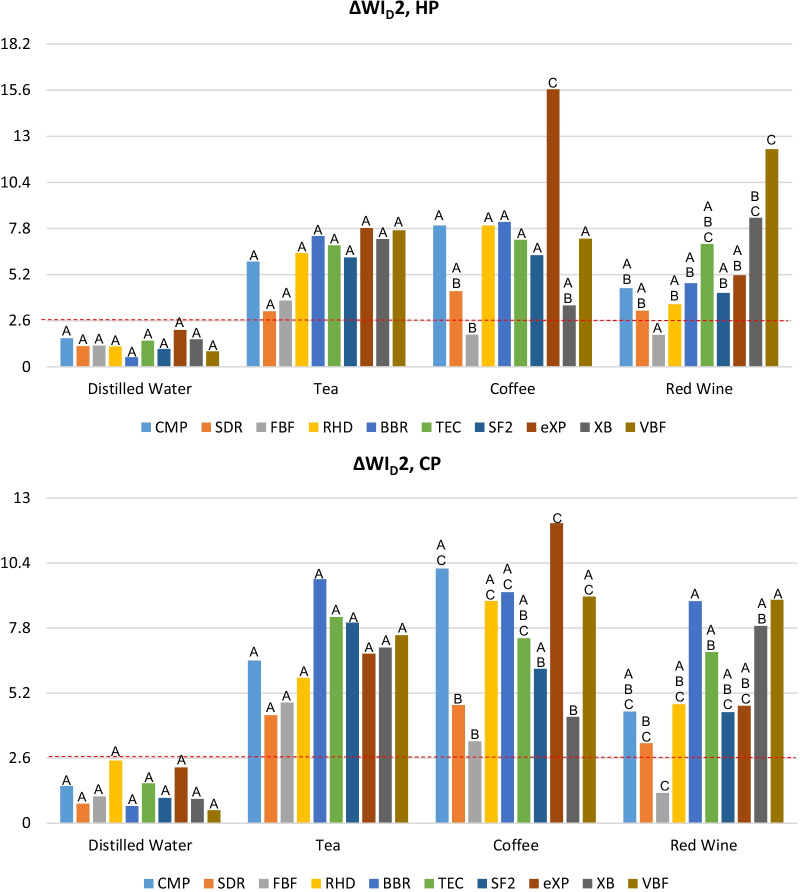
ΔWI_D_2 values of resin composite after two bleaching protocols. The capital letters indicate the difference between the restorative materials. ΔWI_D_2 = WI_D_ (bleaching) − WI_D_ (staining), WPT = 0.72, WAT = 2.60, *CMP* Clearfil Majesty Posterior, *SDR* SDR™ Flow^+^, *FBF* Filtek™ Bulk-Fill Flowable Restorative, *RHD* Reveal HD Bulk, *BBR* Beautifil-Bulk Restorative, *TEC* Tetric EvoCeram® Bulk Fill, *SF2* SonicFill 2, *eXP* everX Posterior™, *XB* X-tra base, *VBF* Venus® Bulk Fill, *HP* hydrogen peroxide, *CP* carbamide peroxide

The mean ΔWI_D_1 and ΔWI_D_2 values and standard deviations of each bleaching group for each resin were shown in Table 3 and the intragroup comparison of the resin composites immersed in each staining solution are schematized in Fig. 3. Regarding ΔWI_D_1 values; intragroup comparisons showed that for all materials, all staining beverages and both bleaching agents, there were no statistically significant difference among the groups (P > 0.05) except for distilled water and coffee groups of eXP (P < 0.05). All values of ΔWI_D_1 were above WPT. ΔWI_D_1 values in all groups of eXP were statistically higher than the ΔWI_D_1 values in all groups of other materials (P < 0.05). Only for CMP, all groups showed acceptable whiteness changes (ΔWI_D_1 < WAT). No imperceptible changes in whiteness (ΔWI_D_1 < WPT) were observed in any of the groups. Regarding ΔWI_D_2 values, no statistically significant difference was detected among the groups of SDR and FBF in terms of staining beverages (P > 0.05). For all restorative materials and all staining beverages, there is no statistically significant difference between the bleaching agents (P > 0.05). The highest ΔWI_D_2 values were observed in eXP group after both bleaching procedures. Clinically acceptable whiteness changes (ΔWI_D_2 < WAT) were observed in all the materials immersed in distilled water. The intragroup comparison of the materials (Fig. 4) subjected to in office bleaching revealed no statistically significant difference between whiteness changes in the CMP, SDR, FBF, and RHD materials immersed in distilled water and those kept in tea and red wine (P > 0.05).

**Table 3:** Mean values ± standard deviations of ΔWI_D_1 and ΔWI_D_2

Material	Solution	ΔWI_D_1		ΔWI_D_2	
		Bleaching method		Bleaching method	
		HP	CP	HP	CP
CMP	Distilled water	1.83 ± 1.29	1.98 ± 1.37	1.62 ± 1.10	1.48 ± 1.05
	Tea	1.96 ± 1.65	1.11 ± 1.05	5.94 ± 4.21	6.50 ± 3.54
	Coffee	1.40 ± 1.7	2.40 ± 1.63	7.97 ± 2.54*	10.18 ± 4.24*
	Red wine	2.07 ± 1.90	1.38 ± 1.40	4.44 ± 2.36	4.46 ± 2.19*
SDR	Distilled water	4.25 ± 1.07	4.58 ± 1.34	1.16 ± 0.60	0.78 ± 0.54
	Tea	3.47 ± 1.44	3.40 ± 0.77	3.13 ± 3.66	4.32 ± 3.15
	Coffee	3.03 ± 1.33	3.10 ± 1.54	4.27 ± 0.77	4.72 ± 1.47
	Red wine	3.15 ± 1.44	2.48 ± 1.29	3.17 ± 1.71	3.20 ± 1.56
FBF	Distilled water	3.26 ± 1.12	3.56 ± 2.31	1.20 ± 0.72	1.07 ± 0.95
	Tea	2.26 ± 0.94	3.83 ± 2.16	3.74 ± 2.62	4.82 ± 2.07
	Coffee	1.20 ± 1.23	1.40 ± 1.10	1.81 ± 1.64	3.27 ± 3.28
	Red wine	1.06 ± 0.75	1.21 ± 0.87	1.80 ± 0.87	1.21 ± 0.88
RHD	Distilled water	4.02 ± 1.26	5.12 ± 1.48	1.15 ± 0.98	2.50 ± 0.90
	Tea	4.47 ± 1.72	4.69 ± 1.60	6.42 ± 2.52	5.81 ± 1.81
	Coffee	3.50 ± 0.82	3.66 ± 2.22	7.97 ± 3.24*	8.88 ± 2.90*
	Red wine	4.02 ± 1.04	3.31 ± 0.86	3.53 ± 0.83	4.76 ± 0.75
BBR	Distilled water	4.34 ± 1.45	4.91 ± 1.15	0.54 ± 0.45	0.69 ± 0.54
	Tea	2.42 ± 1.22	3.14 ± 1.30	7.37 ± 3.50*	9.75 ± 5.26*
	Coffee	1.46 ± 0.69	1.89 ± 1.31	8.18 ± 4.90*	9.23 ± 3.41*
	Red wine	1.90 ± 1.00	2.23 ± 1.47	4.72 ± 2.83	8.87 ± 3.54*
TEC	Distilled water	7.27 ± 1.43	7.26 ± 1.82	1.48 ± 0.55	1.59 ± 0.60
	Tea	7.12 ± 1.06	7.51 ± 2.11	6.86 ± 2.19	8.24 ± 2.89*
	Coffee	5.72 ± 1.19	7.60 ± 1.70	7.17 ± 2.57*	7.39 ± 2.93*
	Red wine	6.25 ± 1.19	7.13 ± 1.75	6.93 ± 1.35*	6.84 ± 1.50
SF2	Distilled water	0.83 ± 0.76	1.37 ± 0.95	1.01 ± 0.48	1.01 ± 0.63
	Tea	3.56 ± 1.03	2.79 ± 0.72	6.17 ± 3.70	8.02 ± 4.28*
	Coffee	2.70 ± 1.54	1.38 ± 1.20	6.30 ± 1.41	6.17 ± 2.32
	Red wine	1.39 ± 1.38	1.73 ± 0.99	4.17 ± 3.49	4.44 ± 2.05
eXP	Distilled water	21.58 ± 4.44	18.51 ± 4.47	2.08 ± 1.15	2.23 ± 1.44
	Tea	21.27 ± 5.49	22.23 ± 4.64	7.83 ± 4.22*	6.77 ± 6.10
	Coffee	20.46 ± 4.43	23.25 ± 2.48*	15.65 ± 2.37*	11.99 ± 4.16*
	Red wine	22.56 ± 3.30	22.29 ± 7.06	5.16 ± 2.16	4.69 ± 2.38
XB	Distilled water	4.24 ± 2.00	4.50 ± 1.19	1.55 ± 0.65	0.97 ± 0.53
	Tea	4.30 ± 2.32	5.83 ± 1.79	7.20 ± 2.53*	7.02 ± 2.94*
	Coffee	2.75 ± 1.33	3.86 ± 1.52	3.47 ± 3.14	4.25 ± 2.94
	Red wine	5.77 ± 1.8	4.51 ± 2.11	8.41 ± 1.07*	7.88 ± 2.87*
VBF	Distilled water	4.82 ± 1.97	3.15 ± 3.70	0.88 ± 0.86	0.52 ± 0.58
	Tea	3.78 ± 2.50	3.08 ± 1.73	7.70 ± 3.99*	7.52 ± 4.20*
	Coffee	4.58 ± 2.33	4.78 ± 2.98	7.23 ± 4.47*	9.06 ± 5.87*
	Red wine	4.34 ± 2.98	4.40 ± 2.38	12.28 ± 5.3*	8.93 ± 5.37*

ΔWI_D_1 = WI_D_ (bleaching) − WI_D_ (baseline). ΔWI_D_2 = WI_D_ (bleaching) − WI_D_ (staining)

WPT = 0.72, WAT = 2.60

*CMP* Clearfil Majesty Posterior, *SDR*: SDR™ Flow^+^, *FBF* Filtek^TM^Bulk-Fill Flowable Restorative, *RHD* Reveal HD Bulk, *BBR* Beautifil-Bulk Restorative, *TEC* Tetric EvoCeram® Bulk Fill, *SF2* SonicFill 2, *eXP* everX Posterior™, *XB* X-tra base, *VBF* Venus® Bulk Fill, *HP* hydrogen peroxide, *CP* carbamide peroxide

*Shows statistically significant difference with the distilled water group for each material (p < .05)

## Discussion

The color change of bulk-fill resin composites which were recently introduced material on the market is of clinical importance in terms of the functional lifetime and esthetic appearance of the restorations. Visual techniques and instrumental techniques can be used to evaluate color differences of restorative materials [29]. It has been stated that visual color assessment is not reliable due to inter-observer inconsistencies in color perception [30]. Therefore, instrumental techniques have been widely preferred for the determination of color changes in dental materials to provide an objective interpretation [31]. In the present study, the clinical spectrophotometer with the CIEDE2000 system which was developed to eliminate the deficiencies in the CIE L*a*b* formula was performed to analyze the color changes of specimens [18]. The CIEDE2000 formula, which was extensively used in previous studies [4, 30], provides better fit compared to CIE L*a*b* formula for calculations of color differences and presents an increased indication of perceptibility and acceptability [32]. In clinical dentistry, visual perceptibility and acceptability threshold values are very important in terms of understanding the color differences in the object [33]. In the current study, color changes were analyzed on the basis of an acceptability threshold of 50:50% (AT:ΔE_00_ = 1.8) and a perceptibility threshold of 50:50% (PT:ΔE_00_ = 0.8) which was reported by Della Bona et al. [33] and Perez et al. [34]. In addition, CIEDE2000 (1:1:1) formula was chosen for the current research due to inadequate data for acceptability and perceptibility thresholds in the CIEDE2000 (2:1:1) formula [17].

Nanohybrid and nanofilled resin composites have been reported to have superior physical and mechanical properties compared to the microfilled and microhybrid resins [35]. In the present study; a nanohybrid resin composite, Clearfil Majesty Posterior was chosen to compare color changes with the bulk-fill resin composites. Although in a previous study [35], it was reported that Clearfil Majesty Posterior exhibited the lowest discoloration than bulk-fill resin composites (SureFil SDR, Ever X, Tetric Evo Ceram), in the current study, the material showed color change regardless of the beverage solutions. Therefore, the first hypothesis of our study, which suggested that “There is no a significant difference between the color stability of the different bulk-fill resin composites compared to the nanohybrid resin composite” is rejected. On the other hand, the mean ΔE_00_ values of the restorative materials used in the study were found as eXP > BBR > CMP > XB > SF2 > VBF > SDR > RHD > TEC > FBF regardless of the staining solutions. Even though the fiber reinforced bulk-fill resin composite showed the highest color change regardless of the solutions in the current study, it was determined that the nanohybrid resin material was prone to discoloration when compared to other bulk-fill resin composites. This is partially consistent with a previous study [3] which investigated the color stability and microhardness of a nanocomposite (Filtek Z350) and 4 bulk-fill composites (Filtek Bulk-Fill, Tetric N-Ceram Bulk-Fill, Sonic Fill-2, and SDR) after immersion in distilled water, tea, coffee, and juice. According to the results of this previous study, Z350 (control group nanocomposite) had the highest ΔE value and SDR had the lowest value.

Regarding staining beverages, previous studies that have examined the discoloration of resin composites have generally used distilled water, cola, coffee, and red wine [36]. The immersion period adopted in this study was 3 h a day at room temperature over a 30-day period, which is equivalent to approximately 5 years of clinical aging, according to Celik et al. [21]. In their pilot evaluation to determine immersion time, the authors reported that the optimal contact time in the mouth between teeth and a beverage was 60 s per cup. The period chosen for the present research simulates 5 years, with an average of 3 cups of beverage consumption per day [21].

In the present study, the specimens immersed in distilled water and red wine showed significantly less color change than coffee and tea regardless of restorative material. These findings mean that the second hypothesis is also rejected. Although the staining effect of distilled water was expected to be the lowest, eXP in distilled water showed statistically higher ΔE_00_ values than coffee and tea. The staining susceptibility of specimens after immersion in distilled water might be due to the degree of water absorption of the material and the hydrophilic/hydrophobic nature of the resin matrix [37]. In some previous studies, artificial saliva has been used as a control group instead of distilled water [38]. Specimens stored in water or artificial saliva have been reported to change color as a result of material aging, but within clinically acceptable limits [39]. However, it has been reported that artificial saliva does not contain intraoral enzymes that cause softening of dimethacrylate polymers on the resin composite surface and hydrolysis of methacrylate ester linkages [38]. In a study [34] that previously tested the efficacy of an in-office bleaching procedure on stained resin composite materials, the researchers used distilled water and artificial saliva as control groups. As a result, it was reported that the color change observed in both control groups was at a clinically acceptable level. Considering these findings, the distilled water was used as a control group in the current study.

Not in consistent with the results of the current research, some studies have found that coffee has a higher discoloration potential than tea [40, 41]. Coffee has a yellow colorant pigment with different polarities which increases the affinity to polymers. It may be the reason for the color change of the resin-based materials [39]. In contrast to the present study, Sayan et al. [36] observed that red wine causes more color change than distilled water, tea, and coffee in a period of 30 days. This variability may be explained by differences in the preparation of staining solutions and the implementation of staining procedures. A previous study [42] emphasized that the mechanisms by which red wine affects color changes in resin composites are the ethanol that it contains, which degrades the organic structure of resin, and alcohol, which causes the absorption of coloring pigments, leading to increased surface roughness [43]. Such effects also arise from the relative acidity (pH = 4.5) and tannin content of red wine, as reported by Ardu et al. [44]. The pH levels of the beverages used as immersion solutions could not be measured in the present study. The lower discoloration of the resin composites in the red wine group could have originated from changes in red wine’s structure given the difficulty in replicating the producer’s storage environment during our test period.

Regarding the ΔE_00_ values after staining in coffee, nanohybrid composite resin and eXP showed statistically higher values in comparison to other resins. eXP also exhibited the statistically highest ΔE_00_ values among the materials immersed in distilled water, tea, and red wine. These findings may be attributed to the fact that eXP has a glass fiber-reinforced structure with a matrix composed of bisphenol A-glycidyl methacrylate (Bis-GMA) [45]. It has been reported that reinforcement with glass fibers may change the color parameters of composite materials since the refractive index of glass-fibers differs from that of the surrounding resin matrix along with its fillers which may favor light penetration through composite [46]. For the fiber-reinforced eXP material, a capping layer with a universal resin composite is recommended because glass fibers prevent good polishing and hinder optimal esthetic clinical results [47]. Inconsistent with the present findings, Tunçdemir et al. [48] has reported no significant difference between the color stability of conventional and fiber-reinforced composite resins after accelerated aging. The reason of inconsistency may be the aging procedure which was applied in the previous study [48]. In connection to this, the lack of an aging process, which is essential for a closer simulation of clinical situations, is a limitation of the present study.

In addition to its fiber-reinforced structure, the Bis-GMA-based matrix of eXP may have an effect on higher ΔE_00_ values after staining procedure. It was reported that the Bis-GMA‐based resin matrix has higher water sorption due to its hydrophilicity which is leading to less stain resistance compared to other methacrylate monomers, such as UDMA [41]. Furthermore, increasing the amount of TEGDMA in the resin matrix from 0 to 1% resulted in the increased water uptake of Bis-GMA‐based resins [41]. In accordance with the results of this study, Barutcugil et al. [4] reported bulk-fill resin composites that include Bis-GMA and TEGDMA, had the highest color change after immersion in beverages compared to nanohybrid resin composite.

The effectiveness of bleaching agents containing peroxide depends on the depth of penetration into the restorative materials, peroxide concentration, and application period [49]. A previous study reported that the concentration and effectiveness of CP are directly proportional [9]. In-office bleaching agents contain high concentration HP formulations as effective ingredients ranging from 35 to 50%. Opalescence PF 16% and Opalescence Boost 40% were preferred in the current study to compare the whiteness effect of at-home and in-office bleaching systems and no difference was found between in-office and at-home bleaching systems in terms of ΔWI_D_ values in all periods. Therefore, the third null hypothesis of our study, which suggested that there is not a significant difference between the bleaching effects of a home and an office bleaching agent, is accepted. Consistent with the results of the present research, a study by Gul et al. [50] compared the bleaching systems (Opalescence Boost 40% and Opalescence PF 15%) on the discolored resin composites and stated that there was no statistically significant difference between the bleaching systems. In contrast, a previous study showed that 10% HP causes more color changes on resin composites than 10% CP [51]. After clinical application, 10% CP is broken down into urea, ammonia, carbon dioxide, and the active ingredient, HP (nearly 3.5%). The HP content of 10% CP is almost three times lower than the HP content in 10% HP, which may have caused the aforementioned result.

As reflected by the ΔWI_D_1 values, both bleaching agents increased the whiteness values of the eXP group for all the staining beverages, even for distilled water. These values are statistically higher than those of all the other resin materials. The changes in whiteness index between the baseline and post-bleaching values were below the clinically acceptable thresholds for CMP. As mentioned earlier, the combination of a resin matrix, randomly orientated E-glass fibers, and inorganic particulate fillers in the eXP structure may have caused the higher ΔWI_D_1 values [52].

The selection of appropriate material for the oral environment is crucial for successful restoration. The storage medium used in the current study could not exactly simulate the oral environment because it contained no saliva, oral microflora, or nutrients, which may also affect the color stability of restorations. Therefore, the results should be supported by additional clinical studies to evaluate the degree of discoloration caused by food and beverages and the effects of bleaching systems on stained resin composites in the oral environment.

## Conclusion

On the basis of the findings and the limitations of an in-vitro evaluation, the findings are summarized as follows: Among the restorative materials evaluated in the study, fiber-reinforced bulk-fill (eXP) and nanohybrid conventional resin composite are more prone to discoloration. In general, coffee and tea exerted more staining effects than distilled water and red wine, except for the eXP group. Although the type of bleaching agent did not affect the color of the stained restorative materials, the nanohybrid composite exhibited a clinically acceptable whiteness change compared to the bulk-fill resin composites. The whiteness changes of the materials after treatment with coffee, tea, and red wine were beyond the clinically acceptable level. Clinicians should be aware that the beverages, in particular, may lead to more visible color change in bulk-fill resins than their conventional counterparts. Dentists may consider postponing composite resin restoration replacements until after vital whitening treatments to ensure optimal esthetic results and should advise their patients in clinics to limit the consumption of coloring solutions such as coffee and tea after bleaching treatments.

## Data Availability

The datasets and materials used or analysed during the current study are available from the lead author on reasonable request.

## Abbreviations

HP: Hydrogen peroxide
CP: Carbamide peroxide
CMP: Clearfil Majesty Posterior
SDR: SDRTM Flow
FBF: FiltekTM Bulk-Fill
RHD: Reveal HD Bulk
BBR: Beautifil-Bulk Restorative
TEC: Tetric EvoCeram® Bulk Fill
SF2: SonicFill 2
eXP: EverX Posterior™
XB: X-tra base
VBF: Venus® Bulk Fill
LED: Light emitting diode
AT: Acceptability thresholds
PT: Perceptibility thresholds
CIELAB: Commission International de l’Eclairage
WI_D_: Whiteness Index for Dentistry
WPT: Whiteness perceptibility threshold
WAT: Whiteness acceptability threshold
ANOVA: Two-way analysis of variance
